# Case Report: A Child With Functional Chronic Duodenal Obstruction Caused by Megaduodenum

**DOI:** 10.3389/fped.2020.585699

**Published:** 2021-01-05

**Authors:** Zhibo Qu, Biao Zheng, Chuncheng Ju, Jiaxu Liu, Bingyang Liu, Haoran Zhang

**Affiliations:** ^1^Department of Pediatric Surgery, The Fourth Affiliated Hospital of Jiangsu University, Zhenjiang, China; ^2^Department of General Surgery, Shenzhen University General Hospital/Shenzhen University Clinical Medical Academy, Shenzhen, China; ^3^Department of General Surgery, Harbin Children's Hospital, Harbin, China

**Keywords:** megaduodenum, duodenal obstruction, pediatric, functional, surgery

## Abstract

Megaduodenum is a clinical syndrome which is characterized by the remarkable expansion of duodenum. Megaduodenum can be caused by mechanical or functional chronic duodenal obstruction. Functional chronic duodenal obstruction of megaduodenum in children is a clinical syndrome characterized by non-mechanical obstruction of the duodenum and marked expansion. It is an extremely rare congenital disease. Our paper report a 1-year-old girl with functional chronic duodenal obstruction caused by megaduodenum.

## Introduction

Megaduodenum is a clinical syndrome which is characterized by the remarkable expansion of duodenum (1). Megaduodenum can be caused by mechanical or functional chronic duodenal obstruction. The disease is usually presented by significant dilation of duodenum ii, iii, and iv. This disease is very rare, and it is rarer in early childhood. Functional chronic duodenal obstruction of mega duodenum in children is a clinical syndrome characterized by non-mechanical obstruction of the duodenum and marked expansion (2). It is an extremely rare congenital disease. Recently, our department treat a related patient with functional chronic duodenal obstruction caused by megaduodenum. The aim of this paper to present the rare case report.

## Case Report

**Figure 1:**
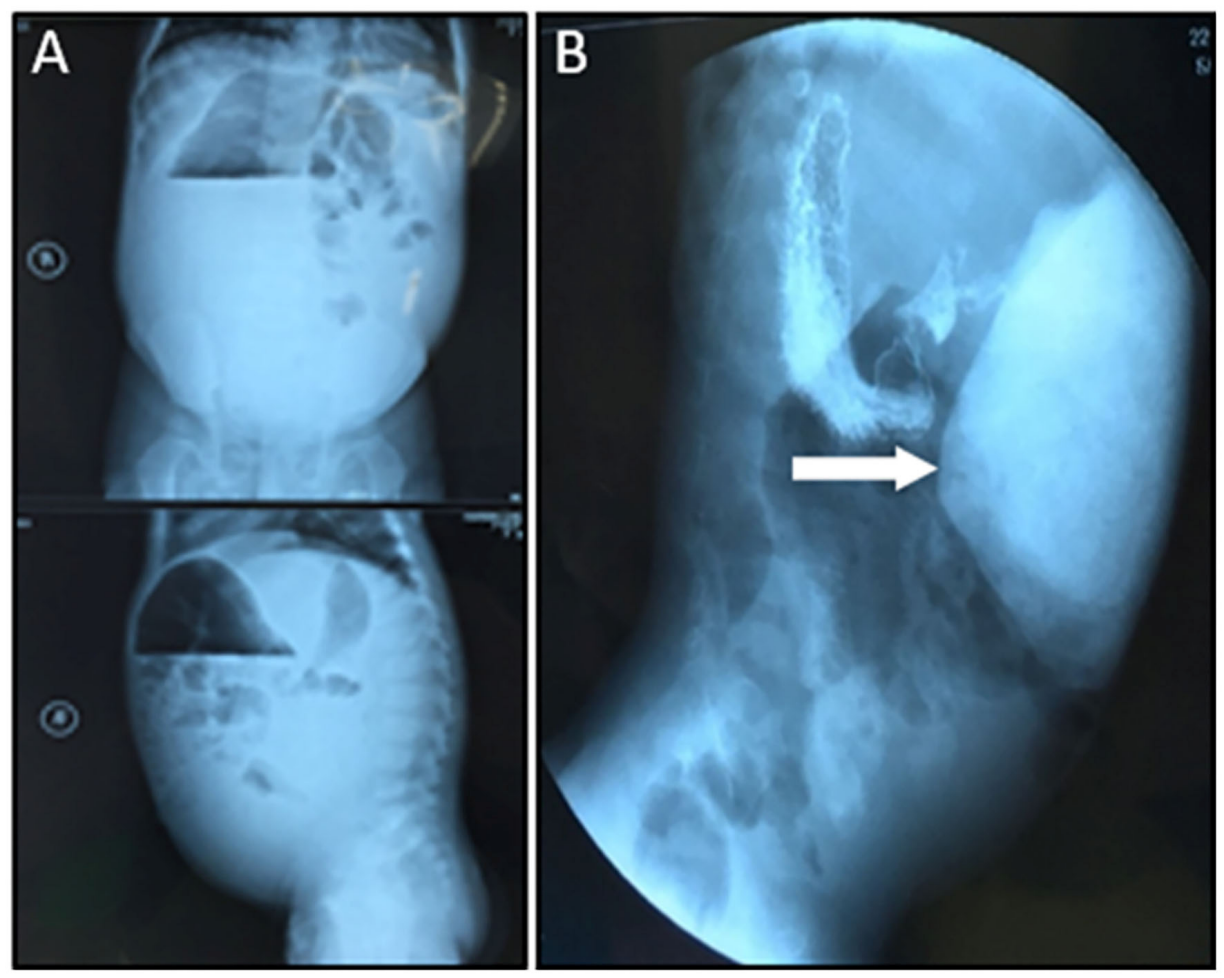
**(A)** Preoperative abdominal X-ray suggested incomplete duodenal obstruction. **(B)** Preoperative UGI suggested the duodenum dilates (White arrow).

**Figure 2:**
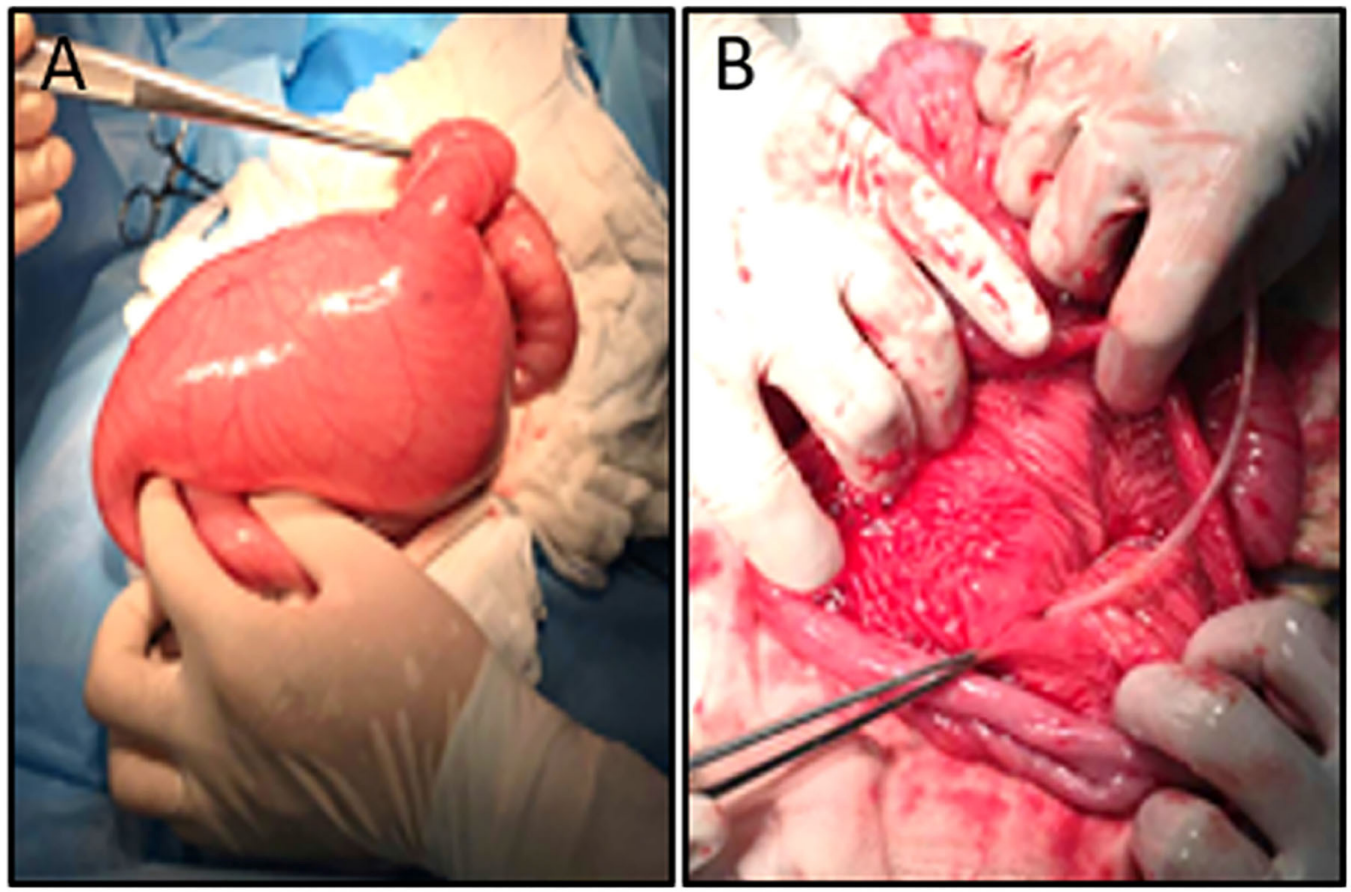
**(A)** Intraoperative demonstrated megaduodenum. **(B)** Protect duodenal ampulla (White arrow).

**Figure 3:**
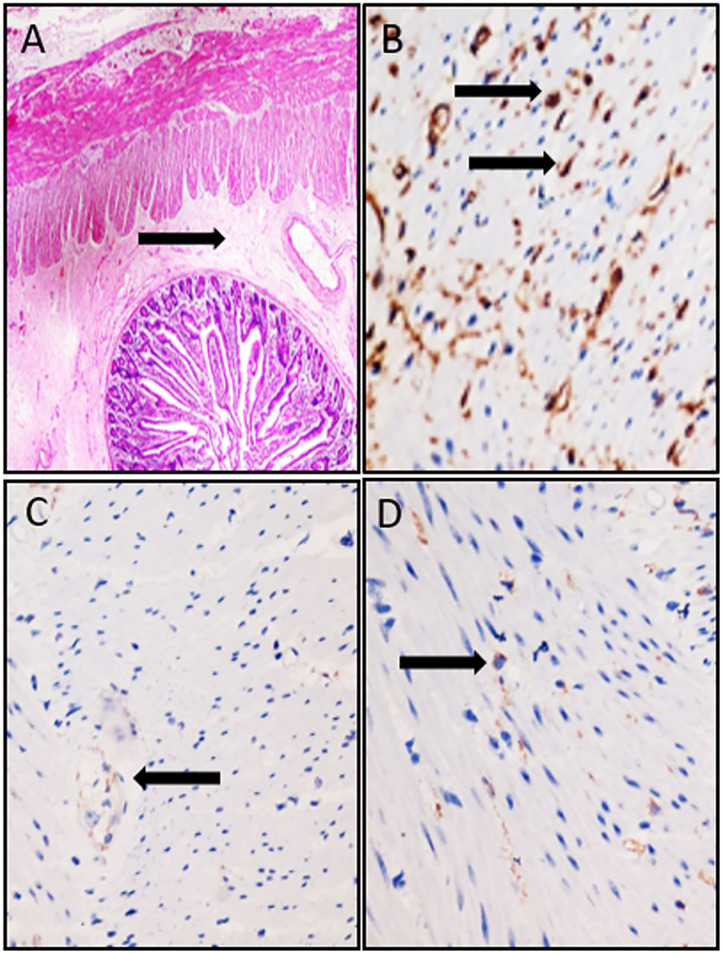
**(A)** HE staining of surgical specimens, the submucosa was loose and edematous (Black arrow, original magnification: 40×). **(B)** IHC staining for CD34. **(C)** IHC staining for CR. **(D)** IHC staining for NSE (Balck arrow indicates a positive appearance, Original magnification: 200×).

**Figure 4:**
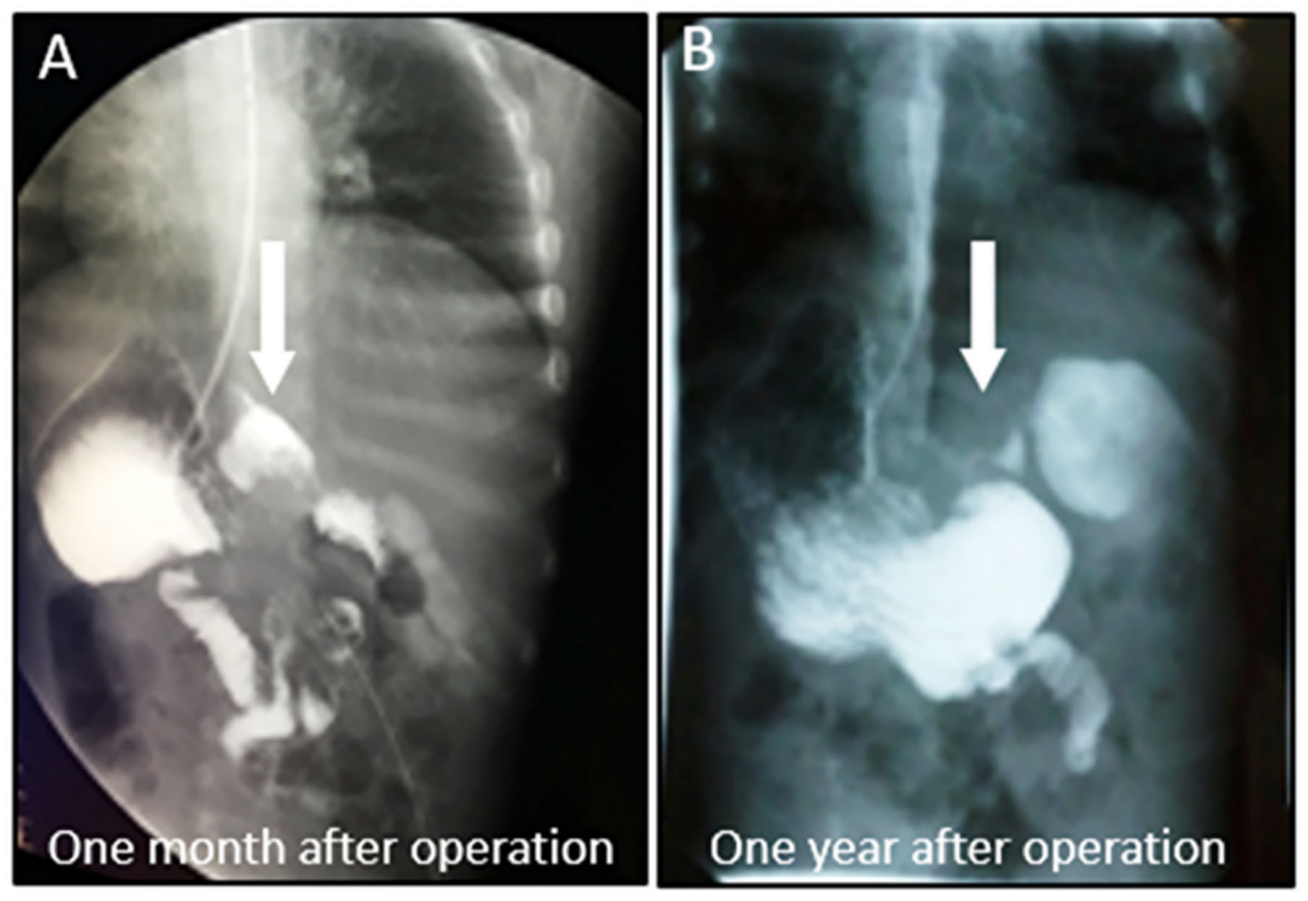
Extended segment extinction, the anastomosis passed smoothly, no narrow. **(A)** Postoperative UGI showed resolution of the megaduodenum (1 month after operation). **(B)** Postoperative UGI showed resolution of the megaduodenum (1 year after operation) (White arrow).

A female patient of 1-year-old was admitted to the hospital with a 5-month abdominal distention. The patient showed gradually increased abdominal distention, abdominal pain and intermittent vomiting. Ask for her medical history, the patient had no history of surgery. Her mother was normal during the pregnancy, and routine prenatal tests did not reveal any significant problems. She was 38 weeks pregnant and delivered by c-section. Admission examination showed abdominal swelling obviously. Accessory examination: vertical abdominal radiograph revealed incomplete intestinal obstruction(Figure 1A); The upper gastrointestinal (UGI) contrast study revealed the duodenum was markedly dilated along its entire length, the size of the expansion about 15 × 12 cm (Figure 1B); Abdominal ultrasound indicates that the duodenum is extremely distal; The patient eventually underwent exploratory laparotomy: the entire duodenum and the beginning of and the beginning of jejunum were markedly dilated, and the diameter was about 15 cm (Figure 2A). There was no obvious mechanical obstruction at the junction between the dilated segment and the jejunum; In the operation, the dilated duodenum was cut off, and pay attention to protect duodenal ampulla (Figure 2B); A loop of jejunum 45 cm from the ligament of Treitz was used for a side-to-side duodenojejunostomy oriented in a retrocolic fashion. Postoperative pathological findings showed that duodenal dilation, intestinal mucosal glands increased, the submucosa was loose and edematous, and the ganglion cells were seen in the muscles (Figure 3A). Immunohistochemistry (IHC): CD34 (+), CR (+), NSE (+), (Figures 3B–D). The patient recovered very well after the operation. Symptoms of bloating and vomiting disappeared. Follow up UGI contrast study after operation showed that the anastomosis passed well without stenosis or obvious reflux and the duodenum was emptying well with near complete resolution of the megaduodenum (Figures 4A,B).

## Discussion

The disease of megaduodenum was first reported by Meichior in 1924. Megaduodenum can be caused by mechanical or functional chronic duodenal obstruction. The mechanical obstructive factors were mainly caused by external compression of duodenal bowel (such as Superior mesenteric artery compression syndrome, annular pancreas, tumor, congenital cord), duodenal intestinal wall (such as Tumor compression, duodenal diverticulum, duodenal inflammation, gastrojejunal postoperative obstruction) and duodenal intestinal obstruction (such as origin of the jejunum is stenosis, atresia, gallstone, fecal calculus, duodenal diaphragm, and parasitic cause) (1). For this patient, during the operation, we found the duodenum was massively dilated along its entire length. We carefully explored the distal duodenum and found no internal stricture or extrinsic compression. So we consider this expansion of duodenum due to functional chronic duodenal obstruction. It is a really rare case. To our knowledge, megaduodenum in children due to functional chronic duodenal obstruction has not been previously reported in the English medical literature. From the limited Chinese medical literature, we can know this type of megaduodenum is likely to be associated with the following factors: genetic factors; lack of inborn nerves in the intestinal nerve, which can lead to intestinal dysfunction and intestinal neurologic dysfunction; multiple myositis and tension myopathy resulting in intestinal myopathy; the combination of intestinal nerve and muscular layer; As a result of scleroderma or amyloidosis, the intestinal wall collagen and the assay lesion are caused (2). The patient postoperative pathological findings showed that duodenal dilation, intestinal mucosal glands increased, the submucosa was loose and edematous, and the ganglion cells were seen in the muscles. We consider the pathological changes in the intestinal muscularis resulting from myositis. IHC results showed that CD34 was positive, which further indicated its inflammatory infiltration. And both NSE and CR were positive, indicating the presence of ganglia. A comprehensive consideration is given that the duodenal plasma muscle layer inflammation may lead to intestinal muscular layer lesions followed by duodenal dilatation. Megaduodenum should be considered as part of the pathophysiology in patients with functional or mechanical proximal gastrointestinal obstruction.

For this patient, in the operation we cut off the dilated duodenum, and made a loop of jejunum 45 cm from the ligament of Treitz was used for a side-to- side duodenojejunostomy oriented in a retrocolic fashion. The patient has recovered well. Zhang proposed that this operation might have drawbacks, and there was still the possibility of recurrence, which should be close the pylorus and made gastric-jejunum Rouen-y anastomosis (3). More aggressive surgical approaches (e.g., radical enterectomy) have been reported for the treatment of hereditary megaduodenum (4). But whatever operation do, the main goals are to relieve or bypass the obstruction which causes the megaduodenum, and improve duodenal emptying and restore GI tract continuity (5). At the same time, we should pay attention to the duodenal papilla, that is, the opening of the common bile duct and duodenum, so as to avoid iatrogenic injury.

## Conclusion

We consider this patient due to functional chronic duodenal obstruction. The patient has recovered well after operation. We consider the pathological changes in the intestinal muscularis resulting from myositis. A comprehensive consideration is given that the duodenal plasma muscle layer inflammation may lead to intestinal muscular layer lesions followed by duodenal dilatation. Functional chronic duodenal obstruction of megaduodenum in children is a clinical syndrome characterized by non-mechanical obstruction of the duodenum and marked expansion. It is an extremely rare congenital disease.

## Data Availability Statement

The original contributions presented in the study are included in the article/supplementary material, further inquiries can be directed to the corresponding author/s.

## Ethics Statement

Written informed consent was obtained from the legal guardian of the participant for the publication of any potentially identifiable images or data included in this article.

## Author Contributions

BZ designed the work, and revised the manuscript. ZQ carried out the study, and prepared the manuscript. CJ, JL, and BL provided clinical data. HZ revised the manuscript. All authors read and approved the final manuscript and agree to be accountable for the content of the work. All authors contributed to the article and approved the submitted version.

## Conflict of Interest

The authors declare that the research was conducted in the absence of any commercial or financial relationships that could be construed as a potential conflict of interest.
